# Enhancement of Herpes Simplex Virus (HSV) Infection by Seminal Plasma and Semen Amyloids Implicates a New Target for the Prevention of HSV Infection

**DOI:** 10.3390/v7042057

**Published:** 2015-04-20

**Authors:** Lilith Torres, Tatiana Ortiz, Qiyi Tang

**Affiliations:** Department of Microbiology, College of Medicine, Howard University, Seeley Mudd Building, Room 315, 520 W Street, NW, Washington, DC 20059, USA; E-Mails: lilithtorres@yahoo.com (L.T.); tatianaot@gmail.com (T.O.)

**Keywords:** herpesvirus, herpes simplex virus (HSV), semen, SEVI (semen-derived enhancer of viral infection) and SEM (semenogelin) amyloids, sexual transmission

## Abstract

Human herpesviruses cause different infectious diseases, resulting in world-wide health problems. Sexual transmission is a major route for the spread of both herpes simplex virus-1 (HSV-1) and -2. Semen plays an important role in carrying the viral particle that invades the vaginal or rectal mucosa and, thereby, initiates viral replication. Previously, we demonstrated that the amyloid fibrils semenogelin (SEM) and semen-derived enhancer of viral infection (SEVI), and seminal plasma (SP) augment cytomegalovirus infection (Tang *et al.*, J. Virol 2013). Whether SEM or SEVI amyloids or SP could also enhance other herpesvirus infections has not been examined. In this study, we found that the two amyloids as well as SP strongly enhance both HSV-1 and -2 infections in cell culture. Along with SP, SEM and SEVI amyloids enhanced viral entry and increased infection rates by more than 10-fold, as assessed by flow cytometry assay and fluorescence microscopy. Viral replication was increased by about 50- to 100-fold. Moreover, viral growth curve assays showed that SEM and SEVI amyloids, as well as SP, sped up the kinetics of HSV replication such that the virus reached its replicative peak more quickly. The interactions of SEM, SEVI, and SP with HSVs are direct. Furthermore, we discovered that the enhancing effects of SP, SEM, and SEVI can be significantly reduced by heparin, a sulfated polysaccharide with an anionic charge. It is probable that heparin abrogates said enhancing effects by interfering with the interaction of the viral particle and the amyloids, which interaction results in the binding of the viral particles and both SEM and SEVI.

## 1. Introduction

Sexually transmitted diseases (STDs) are caused by microorganisms including bacteria, viruses, and protozoa, that colonize the female and male genital tracts, often causing only mild symptoms [1]. STDs affect health and cause social and economic problems worldwide [2,3]. Despite the development of antibiotics and vaccines and the existence of disease prevention and control programs, these pathogens remain important causes of acute and chronic diseases [1]. Most STD pathogens have been detected in semen from asymptomatic and symptomatic men [4]. Viral STD pathogens include human papilloma virus (HPV), human immunodeficiency virus (HIV), herpes simples virus (HSV), hepatitis virus B (HBV), hepatitis virus C (HCV), and cytomegalovirus (CMV), all of which have been detected in semen [4].

HSV infects a large population (over 500 million worldwide) and is associated with a variety of diseases, including genital herpes, most of which cases are caused mostly by HSV-2 and partly by HSV-1 [5]; ocular herpes, induced by HSV-1 and a leading cause of blindness worldwide [5]; and neonatal herpes, which last is usually an infectious consequence of either HSV-1 or HSV-2 via vertical transmission [5,6,7]. One of the characteristics of HSV infection is its ability to latently infect neurons so that they can be reactivated, thereby causing recurrent infections [8]. People who are infected with HSV can shed the virus in their body fluids; for men, those fluids include semen [3]. Viral load in semen is directly related to male-to-female (M-F) transmission of HSV, as well as to the virus’s transmission among men who have sex with men (MSM) [9]. HSV-1 and -2 infections can both be transmitted sexually, with semen being the major carrier of viral particles. As stated above, most cases of sexually transmitted herpes are caused by HSV-2, but in recent years, the number of cases caused by HSV-1 has risen [10]. Although the clinical symptoms of HSV-caused diseases can be controlled with antiviral drugs (acyclovir and valacyclovir), these drugs are not strong enough to stop subclinical transmission [6,11]. No prophylactic or therapeutic vaccine against HSV is available. Topical microbicides that prevent the sexual transmission of viruses could significantly reduce sexually-transmitted diseases. By better understanding the mechanisms employed by HSV in sexual transmission, researchers will be able to design more effective preventive and/or therapeutic drugs against the infection.

To initiate viral infection, the virus and the to-be-infected cell must come into direct contact. The attachment of the virus to the cell can be either specific or non-specific; the type of attachment determines the pathway of viral entry. Viral entry is a critical step of viral transmission. HSV enters the cell via either of two different pathways: the fusion of the viral envelope with the cellular membrane or endocytosis [12]. HSV entry via the fusion of the viral envelope with the plasma membrane requires the specific interaction of the virus with cellular surface receptors, while endocytosis is unspecific [12]. Both pathways require that the virus become attached to the cell surface. Therefore, any enhancement of the attachment of viral particles to cell surfaces will strengthen viral infection, as has been demonstrated in the case of semen, which can enhance HIV and CMV infection [13,14,15].

Semen contains proteolytic cleavage products of prostatic acid phosphatase (PAP) and semenogelins (SEM), each of which can form amyloid fibrils in semen. The first amyloid fibril found to strongly enhance HIV infection is made up of peptide fragments from PAP and was named *semen-derived enhancer of viral infection* (SEVI) [13]. Additional peptides—a second set—that form HIV-enhancing amyloid fibrils are derived from SEM and are referred to as *SEM amyloids* [14]. Both SEVI and SEM have been demonstrated to enhance CMV infection in different cells [15]. The mechanism that SEVI and SEM use to enhance viral infection might be that the intrinsic positive charges of SEVI and SEM facilitate virion attachment to and fusion with target cells because the anionic polymers decrease the enhancement of HIV infection mediated by semen, SEVI, and SEM [14,16]. If the positive charge of the amyloids is the only thing responsible for the enhancement of viral infection, then SEM, SEVI, and SP (semen plasma) should enhance the infection of all viruses with envelopes. To test the presumption, we expanded our investigation to human herpes simplex viruses.

In the present study, we show that SEM and SEVI amyloids as well as SP can enhance both the HSV-1 and the HSV-2 infection of permissive cells and that treating HSV with these substances not only enhances viral particle formation but also increases viral protein production. The enhancement is accomplished by increasing the entry of viruses into the infected cells. These experimental results imply a novel target for preventing HSV infection and, when that has not been or cannot be accomplished, its subsequent spread.

## 2. Results

### 2.1. SEM and SEVI Amyloids and SP Interacted with the HSV Viral Particles

**Figure 1:**
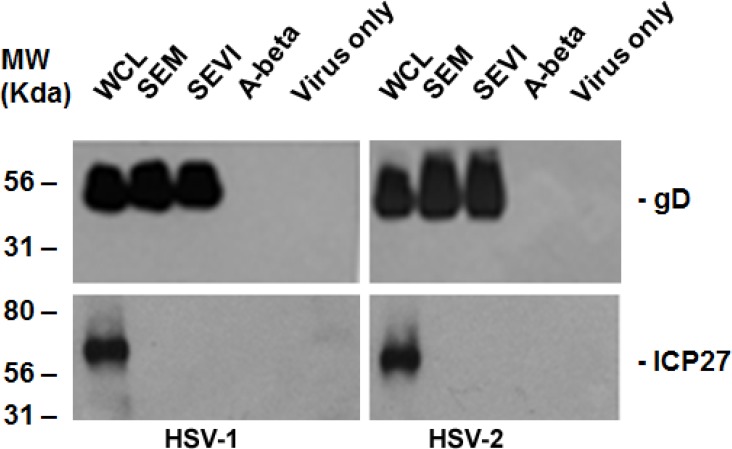
Amyloid-virus binding assay. (**Left**) 1 mL of HSV-1 (10^7^/mL) was incubated with 5 μg of SEVI or SEM amyloid fibrils for 1 h at 37 °C. Controls include virus alone and virus incubated with 5 μg of A-beta amyloid. The mixtures were centrifuged and the pellet was washed twice with MEM. The pellet was lysed in 2× Laemmli buffer and blotted using anti-ICP27 (non-structural protein) and anti-gD (envelope protein) antibodies. Whole cell lysates (WCL) of HSV-1–infected 293T cells were used as input controls for the Western blot; (**Right**) Similar conditions, but using HSV-2 instead of HSV-1. WCL = whole cell lysate made from HSV-infected HEK 293T cells.

As we conducted investigations of the effects of semen on the viral infection, similar to those about which we have previously reported for CMV [15], we were curious about whether semen or semen amyloids could enhance the infections of other sexually transmitted herpesviruses. First we wondered whether HSV-1 and -2 bind to SEVI or SEM. We incubated the purified HSV with SEVI and SEM for 1 h. Controls for the experiment included virus in the absence of amyloids and virus incubated with A-beta (1–42) amyloids. Samples were then centrifuged at 1000 rpm for 5 min on a table-top centrifuge (Eppendorf 5424). The pellets were washed twice with serum-free MEM and resuspended in phosphate-buffered saline (PBS). The resuspended pellets were mixed with the same volume of 2× Laemmli buffer and then subjected to PAGE for Western blotting. As can be seen in Figure 1, the HSV structural protein, glycoprotein D (gD), from the samples of the whole cell lysate (WCL) (WCL was made from HSV-infected HEK 293T cells), SEM (SEM binding) and SEVI (SEVI binding) was positive in the Western blot assay. Non-structural protein ICP27 (infected cell protein 27) can be detected only from samples of WCL. These results demonstrate that both HSV-1 (left panel) and HSV-2 (right panel) can be pulled down by SEM and SEVI, but not by A-beta amyloids.

### 2.2. Viral Protein Production Was Increased by SEM and SEVI Amyloids and by SP

**Figure 2:**
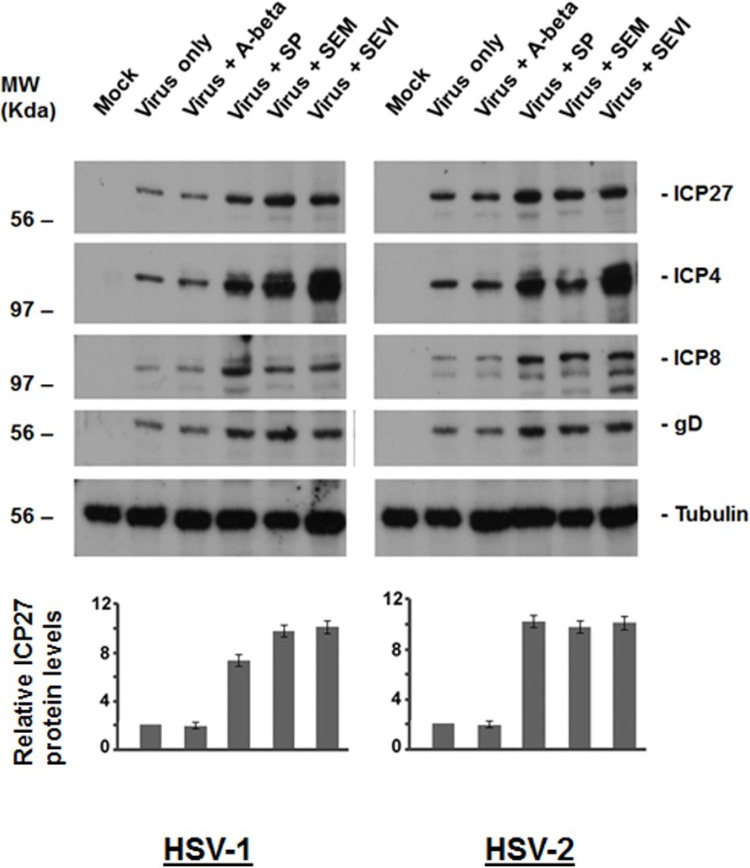
Western blot assay to detect viral protein production. (**Left**) HEK 293T cells were mock-infected or infected with either HSV-1 alone (MOI = 0.1) or HSV-1 treated with SP, A-beta, SEM amyloids, or SEVI for 24 h. Whole cell lysates were prepared for Western blot using antibodies against ICP27, ICP4, ICP8, gD, and tubulin (as loading control). (**Right**) The same as that in the left, but using HSV-2 instead of HSV-1; (**Bottom**) By comparing the density of the ICP27 bands of HSV-1 or HSV-2 with that of the virus alone (with all intensities normalized to the tubulin control), the fold-increases of ICP27 levels for HSV-1 (left) and for HSV-2 (right) were calculated (Quantity One software, version 4.5.0, Bio-Rad Laboratories, Richmond, CA, USA). Normalized ICP27 levels are shown below the corresponding Western blots.

To determine whether SEM, SEVI, and SP could enhance HSV infection, we first examined whether SEM-, SEVI- and SP-treated HSV results in increased viral protein production. In the study, Vero cells were used for infection of HSV-1 and -2, at an MOI of 0.1, and HSV-1 and -2 were treated or untreated as follows (Figure 2): (1) Virus infection alone (that is, virus not exposed to SP or amyloids); (2) virus treated with A-beta (5 μg/mL); (3) virus treated with SP at a dilution of 1:1000; (4) virus treated with SEM amyloids (5 μg/mL); and (5) virus treated with SEVI (5 μg/mL). Twenty-four hours after infection, whole cell lysates were fractionated by SDS-PAGE and analyzed by Western blot using anti-HSV antibodies (Figure 2). The immunoblotted viral proteins include (1) the immediate-early (IE) proteins ICP27 and ICP4; (2) the very early protein ICP8; and (3) late protein gD. Viral protein production was significantly increased by SEM and SEVI amyloids and by SP.

To quantify the relative increase in viral protein production by SEM, SEVI, and SP, we compared the density of the ICP27 bands relative to virus alone and normalized all signals to tubulin. The fold-increase of the ICP27 levels was calculated from three independent experiments and is indicated as shown with a standard error below the corresponding Western blots. The results demonstrate that SEM, SEVI, and SP can increase HSV protein production.

### 2.3. SEVI and SEM Amyloids Increase HSV Infection Rates

Next, we wanted to determine whether SP, SEVI, and SEM could increase HSV infection rates in both Vero and HEK 293T cells. We incubated HSV-1 and -2 with SP or amyloids at 37 °C for 1 h. The untreated HSV-1 and -2 cells were also incubated at 37 °C for 1 h as control. Then, the incubated viruses were used to infect HEK 293T or Vero cells for only 2 h in the presence of a DNA inhibitor (PAA, 400 mg/mL). The cells were washed with PBS, fixed with 1% formaldehyde, and the cell samples used for an immunofluorescent assay so that the infected cells and total cells could be counted using flow cytometry (for the 293T cells) or fluorescent microscope (for the Vero cells). Since gD enters cells along with the infected viral particles, it is used as a marker for viral entry.

**Figure 3:**
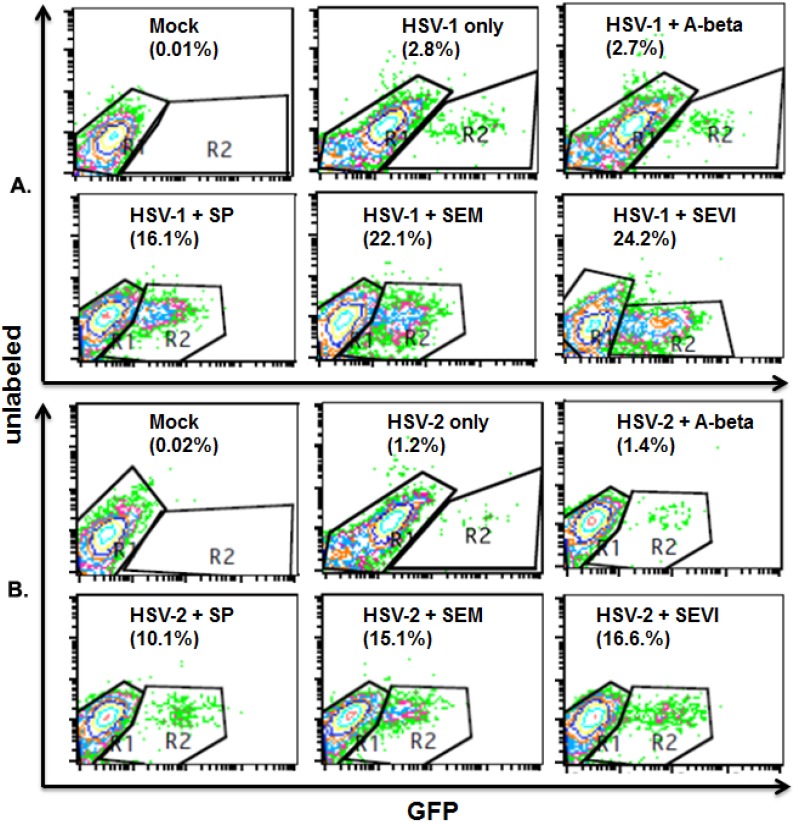
Flow cytometry to detect viral infection rate. (**A**) HSV-1 infection (1 mL per well) in HEK 293T cells. HEK 293T cells were cultured in a 6-well plate. When the cells were 80% confluent, the cells were mock-infected or infected with HSV-1 at an MOI of 0.1. Virus was pre-incubated with 10 μg/mL of A-beta, SEM amyloids, SEVI, or SP (1:1000 dilution) for 1 h at 37 °C before infection. The cells were fixed at 8 h post-infection, stained with anti-gD conjugated with FITC, and measured by flow cytometry to detect the percentage of FITC-positive (infected) cells. The percentage of infected cells is shown in the lower right-hand corner; (**B**) HSV-2 infection in HEK 293T cells. The same experiments as described in A, but using HSV-2 instead of HSV-1. Mock-infected cells in the absence or presence of 10 μg/mL of SEM or 10 μg/mL of SEVI were used to control for autofluorescence. Results are representative of one of three experiments.

For the flow cytometry assay of the 293T cells, we stained cells with anti-gD antibody that was conjugated with FITC. The cell samples were then subjected to flow cytometry to count total cell number and FITC cells (green channel standing for infected cells). As can be seen in the upper panel of Figure 3, without treatment with SP or amyloids or treated with A-beta amyloid, the HSV-1 infection rates are 2.8% and 2.7%, respectively. Treatment with SP, SEM, or SEVI enhanced the HSV-1 infection rate to 16.1%, 22.1%, or 24.2%. The lower panel of Figure 3 shows a similar increase of HSV-2 infection rates attributable to treatment with SP and semen amyloids: The HSV-2 infection rates are 1.2% and 1.4%, respectively, without treatment and with A-beta amyloid treatment. Treatment with SP, SEM, or SEVI increased the HSV-2 infection rate to 10.1%, 15.1%, or 16.6%.

**Figure 4:**
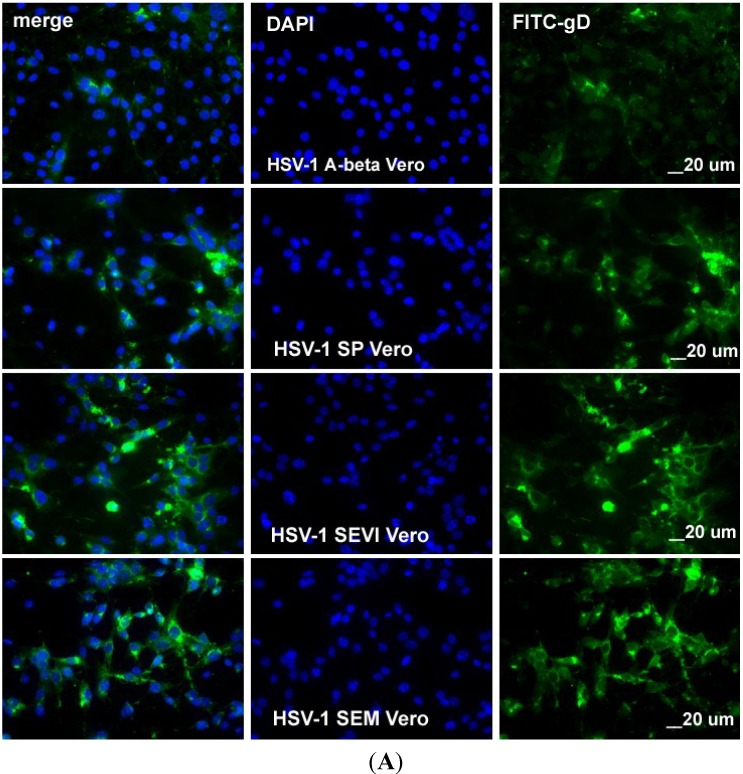

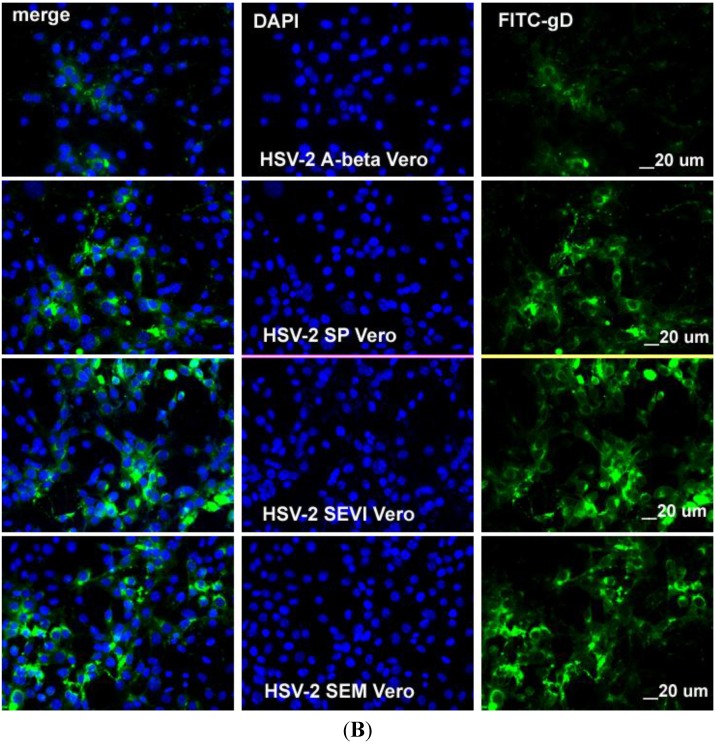
(**A**) Visualization of HSV-1 infection in Vero cells. The Vero cells were infected with HSV-1 at an MOI of 0.5 for 8 h following pre-treatment with either 10 μg/mL of A-beta (first row), 1:1000 SP (second row), SEVI (third row), or 10 μg/mL of SEM (fourth row) amyloids. The cells were fixed with 1% paraformaldehyde and stained with anti-gD-FITC in green and DAPI. The slides were observed under a fluorescence microscope (10× amplification lens), and pictures were taken to show infected cells (green) and total cells (DAPI). All scale bars correspond to 20 μm. The imaging experiments were performed three independent times, and the results as shown are representative of one of three experiments; (**B**) Visualization of HSV-2 infection in Vero cells. Similar to what was done for Figure 4A, except that the Vero cells were infected with HSV-2.

In order to count the total and virus-infected Vero cells under a fluorescent microscope, we stained the cells with anti-gD antibody that was conjugated with FITC and with DAPI (for counting total cells). FITC and DAPI channels were visualized, and the pictures were taken separately. As can be seen in Figure 4A, when the HSV-1 cells were incubated with A-beta amyloid, the infection rate was about 3%, the treatment of HSV-1 with SP, SEVI, or SEM increased the infection rate significantly. A similar result was observed for HSV-2 (Figure 4B). These results are consistent with those found using flow cytometry assays.

### 2.4. Viral Replication was Accelerated and Enhanced by SEM and SEVI Amyloids and by SP

**Figure 5:**
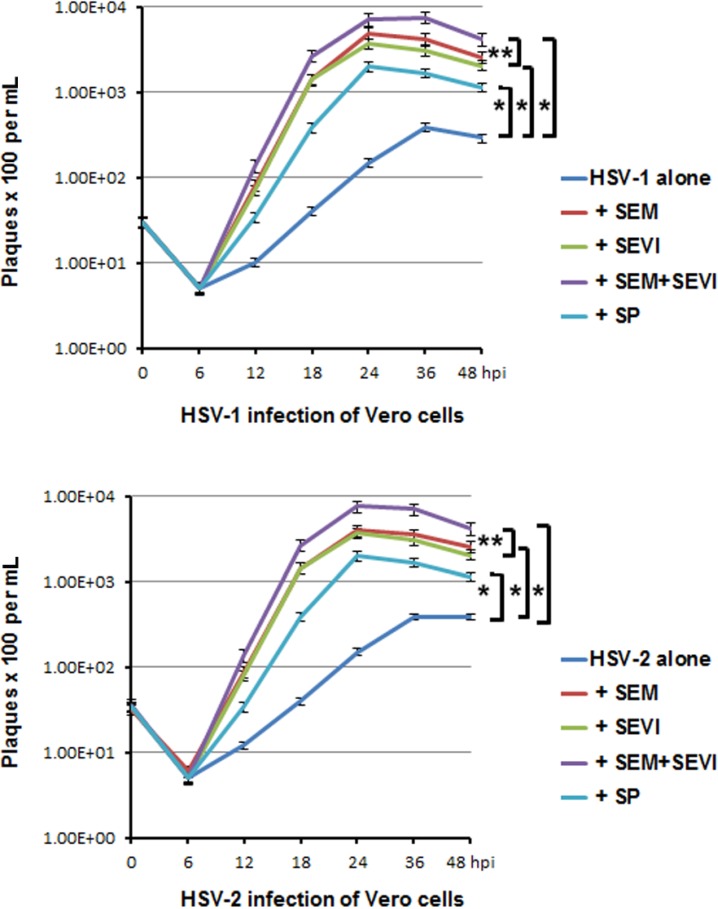
Viral growth curve assay. (**Upper**) HSV-1 infection of Vero cells. Vero cells were infected with virus alone or with virus treated with SP (1:1000 dilution), SEM amyloids (5 μg/mL), SEVI (5 μg/mL), or SEM amyloids (5 μg/mL) plus SEVI (5 μg/mL) at an MOI of 0.1 for a differing number of hours (as indicated). One well of each culture was collected (along with medium) at the indicated hpi and stored at −80 °C. The collected cells (with medium) were frozen/thawed for three cycles and then centrifuged at 8000 g for 20 min to remove the cellular debris. To perform a pfu assay, 25 μL of supernatant of each sample was used to infect Vero cells in triplicate; (**Lower**) the same as described in the above, except that the Vero cells were infected with HSV-2. Student’s *t*-test was used to statistically analyze the differences between the groups *versus* virus alone (*p < 0.001) and those between the group of SEM + SEVI *versus* SEM or SEVI (**p < 0.005).

Lastly, we set up five groups of cultures that were infected with virus (HSV-1 or HSV-2) to measure the extent to which SEVI, SEM, and SP enhance HSV replication in cell culture. The virus was pre-incubated at 37 °C for 1 h with the following: (1) mock treatment (virus alone, no SEM or SEVI); (2) SP (1:1000); (3) SEM amyloids (5 μg/mL); (4) SEVI (5 μg/mL); and (5) 5 μg/mL of SEM + 5 μg/mL of SEVI. The infection of the Vero cells was carried out at an MOI of 0.05. Viral samples containing medium and cells were collected every 6 or 12 h for 48 h to monitor HSV replication by pfu counts (Figure 5). Collected viral samples were frozen/thawed three times to release the viral particles, and centrifuged to pellet cellular debris. Supernatants were assayed for viral production in the Vero cells. As shown in Figure 5, both the HSV-1 (upper) and the HSV-2 (lower) infections were strongly enhanced by a 1:1000 dilution of SP (30-fold for both HSV-1 and -2), 5 μg/mL of SEM amyloids (100-fold for HSV-1 and 80-fold for HSV-2), and 5 μg/mL of SEVI (50-fold for both viruses). We also tested whether the SEM and SEVI amyloids could have a synergistic effect in terms of enhancing HSV infection. Combining SEM and SEVI amyloids (group 5) led to an additive and not a synergistic effect with regard to their enhancing HSV infection.

### 2.5. The Enhancement of SP, SEVI, and SEM in HSV Infections can Be Significantly Blocked by Heparin

**Figure 6:**
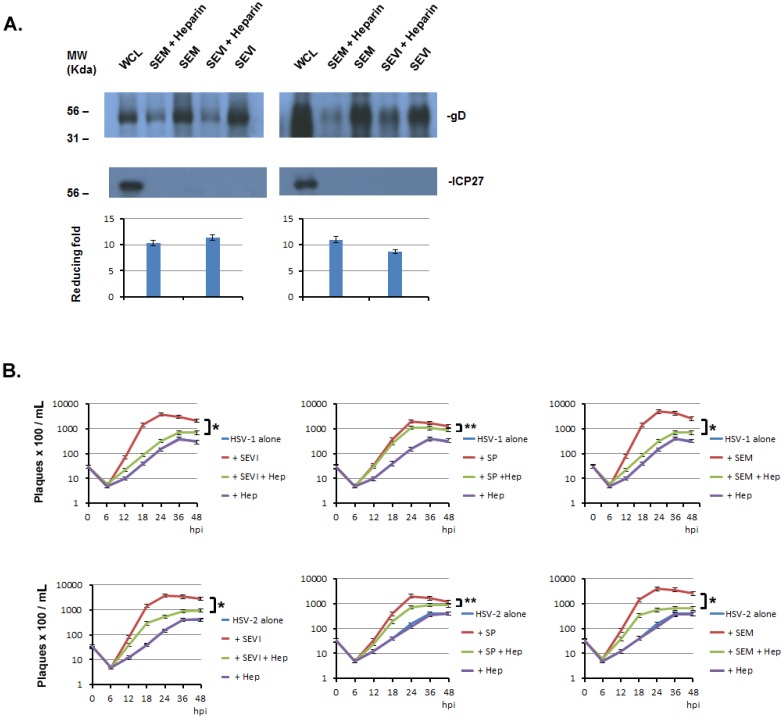
Heparin inhibits the amyloids’ enhancement of HSV infection. (**A**) Heparin interferes with the binding of HSV with amyloids. One milliliter of HSV-1 (10^7^/mL) was incubated with 5 μg of SEVI or SEM amyloid fibrils in the absence or presence of heparin (5 μg/mL) for 1 h at 37 °C. Controls included virus alone and virus incubated with 5 g of A-beta amyloid. The mixtures were centrifuged and the pellet was washed twice with MEM. The pellet was lysed in 2× Laemmli buffer and blotted using anti-ICP27 (non-structural protein) and anti-gD (envelope protein) antibodies. Whole cell lysates (WCL) of HSV-infected 293T cells were used as input controls for the Western blot. The density of the gD bands of HSV-1 or HSV-2 from non-heparin treated was compared with that of treated heparin, the fold increase of gD levels for HSV-1 (left) and for HSV-2 (right) were calculated (Quantity One software, version 4.5.0, Bio-Rad Laboratories, Richmond, CA, USA) and are shown below the corresponding Western blots; (**B**) Heparin significantly reduced the enhancing effects of SEM or SEVI on HSV infection. PFU assays were conducted in Vero cells using the same protocol as that described in Figure 5, except that heparin (5 μg/mL) was used in these experiments. Student’s *t*-test was used to statistically analyze the differences between the group of virus treated with amyloid (SEM or SEVI) *versus* those between virus treated with amyloids plus heparin (Hep) (*p < 0.001) or the differences between the group of virus treated with SP *versus* those between virus treated with SP plus heparin (Hep) (**p < 0.05).

Previous studies have reported that the enhancement of semen amyloids in HIV infection highly depends on their cationic nature, since the enhancing ability can be abrogated by anionic polymers [16]. Heparin is a sulfated polysaccharide with an anionic charge and has been reported to interfere with the interaction of SEVI and SP with HIV virions [16]. We wonder whether it can interfere with the interaction of semen amyloids and HSV virions and whether it can reduce the effects of SP and semen amyloids on HSV infection. We performed an amyloid-virus binding assay with or without heparin. The experimental protocol was the same as that detailed in the legend for Figure 1, except that the heparin was added to the virus and amyloid before mixing the viral solution with amyloids. The pelleted viral particle was subjected to Western blot assay to determine the viral structural protein, gD. As can be seen in Figure 6A, gD band densities are evidently weaker in the heparin-treated groups than are those in the untreated groups. ICP27 is a non-structural protein of HSV and is used to ensure that the detected gD comes from viral particles. Therefore, heparin can significantly reduce the binding of virus with amyloids. We calculated the reducing fold by heparin from three independent amyloid-virus binding experiments by comparing the density of gD band from heparin-treated group with that from untreated group. The averaged reducing fold was shown in the Figure 6A (bottom).

Next, we wondered whether the inhibitory effect of heparin on amyloid-virus binding would cause a decreased enhancement of amyloids in HSV infection. To test this, we infected Vero cells with HSV alone, with HSV that was treated with amyloid (SEM or SEVI) or SP, with HSV that was treated with heparin and amyloid (or SP), or with HSV that was treated with heparin. A viral plaque forming unit (PFU) assay was used to determine the viral growth curve. Six groups of assays are shown in Figure 6B; the averaged PFU per mL was calculated from three independent experiments. First, the heparin alone had no effect on viral entry and viral replication (the HSV alone *versus* HSV heparin), suggesting that heparin has no significant effect on HSV infection in our experimental system; second, heparin appears to significantly reduce the enhancing effects of SEM, SEVI, and SP on HSV infection when the virus and the amyloids or the SP are treated with heparin before infection; last, heparin’s inhibitory effects on SEM or SEVI are apparently greater than those on SP are.

## 3. Discussion

All of the currently defined viral STD pathogens have been detected in semen [4,17]. Therefore, semen may be an important carrier in terms of viral sexual transmission. Of the eight human herpesviruses, four have been defined as STD pathogens, and they are EBV, CMV, HSV-1, and HSV-2 [18]. HSV is one of the most common viruses, occurring in all human populations [4], and genital HSV includes two distinct but closely related viruses: HSV-1 and HSV-2. HSV-1 causes oral and, occasionally, genital sores, while HSV-2 is the most common cause of genital herpes, which can be asymptomatic at the time of primary, initial, or recurrent infection [19].

Human semen contains cationic amyloid fibrils that are aggregated from a wide variety of soluble proteins and peptides [20,21]. These amyloid fibrils share certain properties, including self-assembly by a nucleation-dependent process and a non-branching fibrillar cross-β structure with β strands perpendicular to the fibril axis [21,22]. Pioneering studies have revealed that two amyloid fibrils, SEVI and SEM, are linked to viral infection. SEVI is derived from prostatic acidic phosphatase (PAP) and SEM from semenogelin. Both SEVI and SEM were found to strongly enhance CMV and HIV-1 infections [13,14,15]. In this report, we expand our investigations to determine whether semen plasma and semen amyloids can enhance the infection and replication of HSVs.

The fluid on the surface of the mucosa of the genital track contains different kinds of materials that act against viral infection, especially against viral entry [23]. A high viral load and/or viral absorption on the genital mucosa are important for the success of viral infection. As we expect, SP and the semen amyloids (SEVI and SEM) were found to be able to enhance HSV replication through enhancing viral entry. The semen amyloids can directly interact with viral particles, as can be observed using the virus-amyloid binding assay (Figure 1), which might result in multiple viral particles aggregating on an amyloid to more easily colonize on the surface of genital mucosa. This speculation is confirmed by our experimental studies (shown in Figure 3 and Figure 4), which showed that semen plasma and both amyloids enhanced the HSV entry. The enhancement of viral entry accordingly strengthened viral gene expression (Figure 2) and viral replication (Figure 5). The results we obtained from cell culture experiments support the theory that semen acts as a vector for HSV’s sexual transmission, not only carrying infectious viral particles but also providing an environment that is beneficial for HSV infection. However, our current studies are only at the initial stage. In the future, it is necessary to test the enhancing effects of semen on clinical strains of HSV in different cells (including vaginal epithelial cells).

Developing novel strategies against HSV infection is a global public health priority. Three ways exist to fight HSV infection: inhibiting viral replication by anti-HSV drugs, killing or inactivating infectious viral particles using microbicides, and preventing viral infection with vaccines. Prevention is better than a cure, as antiviral drugs do not destroy their target pathogen; instead they inhibit their development. A novel strategy for HSV prevention is the development of mucosally delivered microbicides. In the case of semen-mediated viral transmission, developing compounds to interfere with the amyloids’ abilities to enhance viral entry might be significant in preventing HSV infection in the general population. Several chemicals have been used in preclinical trials to antagonize viral transmission via semen. The anionic polymer PRO-2000 showed robust activity in preclinical studies [1,18] but failed to show efficacy in an MDP 301 trial [24], possibly because its antiviral activity is inhibited by seminal plasma. New drugs are needed. Our findings that semen components strongly enhance viral infection and that SEM and SEVI protect CMV from being neutralized by anti-gB led us to investigate the extent to which antibodies against SEM, SEVI, and viral surface antigens can inhibit the semen-mediated enhancement of CMV infection in cell culture and in organ culture. These novel microbicide agents (antibodies) are expected to be able to avoid the side effects and toxicities because the antibodies will specifically interact with molecules in seminal fluid. The concept is also applicable to other sexually transmissive viruses, such as HSV-1 and -2. After this phase of the mechanistic studies of the effects of semen, SEVI, and SEM on herpes viral infection, we expect to continue our research with pre-clinical and/or clinical studies exploring how to prevent the sexual transmission (specifically, intercourse) of HCMV and HSV.

## 4. Materials and Methods

### 4.1. Tissue Culture and Viruses

Vero (ATCC, CCL-81, USA) and HEK293T (ATCC, USA) cells were used for the infection of HSV-1 and HSV-2. Vero cells were used because they adhere well to coverslips for immunofluorescent assays. HEK 293T cells were used because they are easily detached from cell culture dishes and facilitate flow cytometry assays. All cells were maintained in Dulbecco's Modified Eagle’s medium (DMEM) supplemented with 10% fetal calf serum (FCS) and 1% penicillin-streptomycin (PS). Wild-type HSV-1 17 was obtained from R.D. Everett and used previously [25]. HSV-2 strain G was purchased from ATCC (VR-734). For immunohistochemical staining, cells were grown on round coverslips (Corning Incorporated, Corning, NY, USA) in 24-well plates (Falcon; Becton Dickinson Labware, Lincoln Park, NJ, USA).

### 4.2. Reagents

SEVI was synthesized by the genomic and the proteomics core laboratories at the University of Pittsburgh (PA, USA). SEM amyloids (amino acid 49–107) [14] were synthesized by Celtek Peptides and provided by Dr. Roan (University of California at San Francisco, USA). Synthetic peptides were dissolved in PBS or serum-free MEM and agitated overnight at 1400 rpm at 37 °C (using an Eppendorf Thermomixer), as described previously [13]. A-beta (1–42) amyloids (Sigma, cat. #A9810) were purchased from Sigma–Aldrich (St. Louis, MO, USA). Semen samples were obtained from 10 different individuals and mixed. The pooled semen samples were centrifuged at 700 g for 5 min, and the supernatant (seminal plasma) was stored at −80 °C and for later use in the experiments. The obtaining and use of semen samples and the informed consent forms were fully reviewed and approved by the committee on human research at Ponce Health Sciences University before the initiation of the study (IRB number 081106-YY). Viral polymerase inhibitor phosphonoacetic acid (PAA) was purchased from Sigma–Aldrich (St. Louis, MO, USA).

### 4.3. Antibodies

The antibodies used for Western blot (WB) and immunofluorescence are listed below. A monoclonal antibody against tubulin (T-9026) was purchased from Sigma–Aldrich (St. Louis, MO, USA; 1:1000 for WB); polyclonal antibody against ICP8, and monoclonal antibodies against HSV ICP4, ICP27, and gD were purchased from Santa Cruz Biotechnology, Inc. (Santa Cruz, CA, USA).

### 4.4. Purification of Viruses

Herpesviruses were amplified in Vero cells. The viral supernatant was centrifuged at 8000 g for 20 min to remove cell debris. The clarified medium was transferred into SW27/28 ultra-clear centrifuge tubes that were underlain with 7 mL of 20% sorbitol buffer (20% D-sorbitol, 50 mM Tris-HCl, pH 7.2, and 1 mM MgCl) and centrifuged at 55,000 g for 1 hour. The purified viral pellet was resuspended in PBS.

### 4.5. Amyloid Fibril-Virus Binding Assay

Amyloid fibrils are large structures and can be pelleted by low-speed centrifugation (e.g., 1000 rpm on a table centrifuge) [16,26]. Purified viral particles (HSV-1 and -2) were incubated with 50 μg of SEVI- or SEM-derived amyloid fibrils at 37 °C for 1 h. Controls for the experiment included virus in the absence of amyloids and virus incubated with 50 μg/mL of A-beta (1–42) amyloids (Sigma, cat. #A9810). Samples were then centrifuged at 1000 rpm for 5 min on a table-top centrifuge (Eppendorf 5424). The pellets were washed twice with serum-free MEM and resuspended in phosphate-buffered saline (PBS). The resuspended pellets were mixed with the same volume of 2× Laemmli buffer. After a 5 min heat treatment at 95 °C, the proteins were separated by sodium dodecyl sulfate-polyacrylamide gel electrophoresis (SDS-PAGE) and analyzed by Western blot, as described below.

### 4.6. Immunoblot Analysis

Proteins were separated by SDS-PAGE (10 to 20 µg loaded in each lane), transferred to nitrocellulose membranes (Amersham Inc., Piscataway, NJ, USA), and blocked with 5% nonfat milk for 60 min at room temperature. Membranes were incubated overnight at 4 °C with primary antibody, followed by incubation with a horseradish peroxidase-coupled secondary antibody and detection with enhanced chemiluminescence (Pierce, Rockford, IL, USA), according to standard methods. Membranes were stripped with stripping buffer (100 mM 2-mercaptoethanol, 2% SDS, 62.5 mM Tris-HCl, pH 6.8), washed with PBS-0.1% Tween-20, and used to detect additional proteins.

### 4.7. Cell visualization by Confocal Microscopy

To visualize HSV-infected cells, the cells were seeded on coverslips and, 12 hours after infection, washed twice with phosphate-buffered saline (PBS), fixed in 1% paraformaldehyde for 10 min at room temperature, permeabilized with 0.2% triton-X 100 in ice for 20 min, and washed 2 more times with PBS. Then the cells were incubated with anti-ICP4 antibody and Texas Red (TR)-conjugated secondary antibody and stained with DAPI. Cells were examined at 10× magnification with a Leica TCS SPII confocal laser scanning system equipped with a water-cooled argon-krypton laser. Two channels (DAPI and TR) were recorded sequentially. DAPI staining was used to show the total number of cells recorded.

### 4.8. Flow Cytometry

For quantitation of infection efficiency, cells were infected for 12 h with viruses, trypsinized, and fixed in 1% paraformaldehyde. The cells were then stained using anti-ICP4 antibody (described as above) and analyzed by a FACSCalibur system with 2 lasers and 4 channels (BD Biosciences, MA, USA) to detect total cell numbers and cells with TR fluorescence. Mock-infected cells with or without treatment with amyloids served as autofluorescence controls.

### 4.9. Plaque Formation Unit (pfu) Assay

Viral titers were determined by the plaque assay, essentially as described [27], but with some slight modifications. Supernatants containing serially diluted viral particles (total volume was 1 mL for each well) were added to confluent Vero cell monolayers in 6-well plates. After adsorption for 2 h, the medium was removed and the cells were washed twice with serum-free DMEM and overlaid with phenol-free DMEM containing 5% FCS, 0.5% low-melting point agarose (GIBCO), and 1% penicillin-streptomycin. Then, the number of plaques that were stained red was counted and reported as pfu per mL. Mean pfu was determined after averaging the number of pfu from different dilutions. Student’s *t*-test was used to statistically analyze differences between the groups; a *p*-value lower than 0.005 was used as the threshold for a significant difference.

## References

[1] Ochsendorf F.R. (2008). Sexually transmitted infections: Impact on male fertility. Andrologia.

[2] Muvunyi C.M., Masaisa F., Bayingana C., Mutesa L., Musemakweri A., Muhirwa G., Claeys G.W. (2011). Decreased susceptibility to commonly used antimicrobial agents in bacterial pathogens isolated from urinary tract infections in Rwanda: Need for new antimicrobial guidelines. Am. J. Trop. Med. Hyg..

[3] Garolla A., Pizzol D., Bertoldo A., Menegazzo M., Barzon L., Foresta C. (2013). Sperm viral infection and male infertility: Focus on HBV, HCV, HIV, HPV, HSV, HCMV, and AAV. J. Reprod. Immunol..

[4] Gimenes F., Souza R.P., Bento J.C., Teixeira J.J., Maria-Engler S.S., Bonini M.G., Consolaro M.E. (2014). Male infertility: A public health issue caused by sexually transmitted pathogens. Nat. Rev. Urol..

[5] Chentoufi A.A., Benmohamed L. (2012). Mucosal herpes immunity and immunopathology to ocular and genital herpes simplex virus infections. Clin. Dev. Immunol..

[6] Shin H., Iwasaki A. (2013). Generating protective immunity against genital herpes. Trends Immunol..

[7] Hadigal S., Shukla D. (2013). Exploiting herpes simplex virus entry for novel therapeutics. Viruses.

[8] Diefenbach R.J., Miranda-Saksena M., Douglas M.W., Cunningham A.L. (2008). Transport and egress of herpes simplex virus in neurons. Rev. Med. Virol..

[9] Garland S.M., Tabrizi S.N. (2004). Diagnosis of sexually transmitted infections (STI) using self-collected non-invasive specimens. Sex Health.

[10] Looker K.J., Garnett G.P., Schmid G.P. (2008). An estimate of the global prevalence and incidence of herpes simplex virus type 2 infection. Bull World Health Organ..

[11] Johnston C., Saracino M., Kuntz S., Magaret A., Selke S., Huang M.L., Schiffer J.T., Koelle D.M., Corey L., Wald A. (2012). Standard-dose and high-dose daily antiviral therapy for short episodes of genital HSV-2 reactivation: Three randomised, open-label, cross-over trials. Lancet.

[12] Bernard Roizman D., Richard M.K., Whitley J. (2007). The family Herpesviridae: A Brief Introduction.

[13] Munch J., Rucker E., Standker L., Adermann K., Goffinet C., Schindler M., Wildum S., Chinnadurai R., Rajan D., Specht A. (2007). Semen-derived amyloid fibrils drastically enhance HIV infection. Cell.

[14] Roan N.R., Muller J.A., Liu H., Chu S., Arnold F., Sturzel C.M., Walther P., Dong M., Witkowska H.E., Kirchhoff F. (2011). Peptides released by physiological cleavage of semen coagulum proteins form amyloids that enhance HIV infection. Cell Host Microbe..

[15] Tang Q., Roan N.R., Yamamura Y. (2013). Seminal Plasma and Semen Amyloids Enhance Cytomegalovirus Infection in Cell Culture. J. Virol..

[16] Roan N.R., Munch J., Arhel N., Mothes W., Neidleman J., Kobayashi A., Smith-McCune K., Kirchhoff F., Greene W.C. (2009). The cationic properties of SEVI underlie its ability to enhance human immunodeficiency virus infection. J. Virol..

[17] Kaspersen M.D., Hollsberg P. (2013). Seminal shedding of human herpesviruses. J. Virol..

[18] Kapranos N., Petrakou E., Anastasiadou C., Kotronias D. (2003). Detection of herpes simplex virus, cytomegalovirus, and Epstein-Barr virus in the semen of men attending an infertility clinic. Fertil. Steril..

[19] Wald A., Zeh J., Selke S., Warren T., Ryncarz A.J., Ashley R., Krieger J.N., Corey L. (2000). Reactivation of genital herpes simplex virus type 2 infection in asymptomatic seropositive persons. N. Engl. J. Med..

[20] Chiti F., Dobson C.M. (2006). Protein misfolding, functional amyloid, and human disease. Annu. Rev. Biochem..

[21] Jarrett J.T., Lansbury P.T. (1993). Seeding "one-dimensional crystallization" of amyloid: A pathogenic mechanism in Alzheimer's disease and scrapie?. Cell.

[22] Sunde M., Blake C. (1997). The structure of amyloid fibrils by electron microscopy and X-ray diffraction. Adv. Protein. Chem..

[23] Uyangaa E., Patil A.M., Eo S.K. (2014). Prophylactic and therapeutic modulation of innate and adaptive immunity against mucosal infection of herpes simplex virus. Immune Netw..

[24] Pirrone V., Wigdahl B., Krebs F.C. (2011). The rise and fall of polyanionic inhibitors of the human immunodeficiency virus type 1. Antiviral. Res..

[25] Tang Q., Li L., Ishov A.M., Revol V., Epstein A.L., Maul G.G. (2003). Determination of minimum herpes simplex virus type 1 components necessary to localize transcriptionally active DNA to ND10. J. Virol..

[26] Yolamanova M., Meier C., Shaytan A.K., Vas V., Bertoncini C.W., Arnold F., Zirafi O., Usmani S.M., Muller J.A., Sauter D. (2013). Peptide nanofibrils boost retroviral gene transfer and provide a rapid means for concentrating viruses. Nat. Nanotechnol..

[27] Martinez F.P., Cosme R.S., Tang Q. (2010). Murine cytomegalovirus major immediate-early protein 3 interacts with cellular and viral proteins in viral DNA replication compartments and is important for early gene activation. J. Gen. Virol..

